# Lower hippocampal volumes at baseline are associated with higher volume loss in healthy elderly

**DOI:** 10.3389/fnagi.2025.1542857

**Published:** 2025-07-16

**Authors:** Vivian Schultz, Benita Schmitz-Koep, Aurore Menegaux, Melissa Thalhammer, Severin Schramm, Su Hwan Kim, Claus Zimmer, Christian Sorg, Panteleimon Giannakopoulos, Marie-Louise Montandon, François R. Herrmann, Cristelle Rodriguez, Sven Haller, Dennis M. Hedderich

**Affiliations:** ^1^Department of Diagnostic and Interventional Neuroradiology, School of Medicine and Health, TUM Klinikum Rechts der Isar, Technical University of Munich, Munich, Germany; ^2^TUM-NIC Neuroimaging Center, School of Medicine and Health, Technical University of Munich, Munich, Germany; ^3^Department of Psychiatry, School of Medicine and Health, TUM University Hospital, Technical University of Munich, Munich, Germany; ^4^Department of Psychiatry, Faculty of Medicine, University of Geneva, Geneva, Switzerland; ^5^Division of Institutional Measures, Medical Direction, Geneva University Hospitals, Geneva, Switzerland; ^6^Department of Surgical Sciences, Neuroradiology, Uppsala University, Uppsala, Sweden; ^7^CIMC - Centre d’Imagerie Médicale de Cornavin, Geneva, Switzerland; ^8^Department of Surgical Sciences, Radiology, Uppsala University, Uppsala, Sweden; ^9^Faculty of Medicine, University of Geneva, Geneva, Switzerland; ^10^Department of Radiology, Beijing Tiantan Hospital, Capital Medical University, Beijing, China

**Keywords:** brain, MRI, hippocampus, atrophy, neurodegeneration, aging

## Abstract

**Introduction:**

Hippocampal volume loss occurs physiologically with age, but an accelerated rate of volume loss is linked to neurodegenerative diseases. While evidence suggests that cross-sectional study designs tend to underestimate hippocampal atrophy rates compared to longitudinal approaches, few studies have directly examined the relationship between these two methods in the context of brain aging. This study aims to investigate the association between baseline hippocampal z-scores and hippocampal volume loss over time in a cohort of healthy older adults.

**Methods:**

182 healthy elderly subjects (mean age: 73.4 ± 3.5 years) who underwent structural Magnetic resonance imaging (MRI) at two timepoints (mean time between the scans 4.8 ± 1.0 years) were included. A subset of participants (*n* = 103) also completed Positron emission tomography (PET) amyloid imaging. Hippocampal volumes were measured at baseline and follow-up using FreeSurfer (v7.1.1). Baseline volumes were adjusted for age and intracranial volume (ICV) and converted into z-scores. The annualized percent change (APC) in hippocampal volume was calculated for each participant. Neuropsychological assessments were conducted at baseline, 18, and 54 months, and APOE genotyping was performed. Correlation analyses examined the relationship between baseline hippocampal volumes and APC, while multiple regression models explored potential influencing factors.

**Results:**

Hippocampal volumes decreased from baseline to follow-up [mean APC (SD): right −1.34% (0.94), left: −1.79% (1.00)]. Small, but statistically significant positive correlations were found between baseline hippocampal z-scores and APC of hippocampal volumes over time, indicating that the lower the volume at baseline, the greater the atrophy rate to timepoint two (right hippocampus: *r* = 0.17, *p* = 0.01; left hippocampus: *r* = 0.14, *p* = 0.03). No covariates significantly influenced this association (*p* > 0.05).

**Conclusion:**

Lower baseline hippocampal z-scores are associated with a greater rate of hippocampal atrophy to the follow-up examination. If validated in larger cohorts, these findings could help establish cut-off values for pathological atrophy in cross-sectional studies.

## 1 Introduction

Normal aging is associated with decreases in brain volume. However, the transition from normal decreases to pathologic neurodegeneration occurs steadily (Jack et al., 1998).

Temporal lobe structures, such as the hippocampus, are of specific interest when studying aging due to their pivotal role in memory function and their early and severe involvement in the neurofibrillary pathology of Alzheimer’s disease (AD) (Hyman et al., 1984; Frankó et al., 2013). Both longitudinal and cross-sectional study approaches have identified a decline in hippocampal volumes among individuals with AD, mild cognitive impairment (MCI) and healthy individuals, with AD exhibiting an accelerated rate of atrophy (Jack et al., 1998; Henneman et al., 2009; Shi et al., 2009). More importantly, a study among 1209 cognitively normal individuals revealed that hippocampal atrophy may precede abnormal amyloid Positron emission tomography (PET) findings, underscoring the significance of preexisting structural deficits linked to aging (Jack et al., 2015).

Understanding the structural aging process is essential for distinguishing normal age-related atrophy from pathological decline. Several studies have examined typical brain aging, most of these studies are based on cross-sectional data (Raz et al., 2004). While cross-sectional studies are cost-effective and in general allow for great sample sizes, they also come with several limitations. One major issue is the variation in normal cerebral morphology and anatomical variance among individuals, which makes it more challenging to accurately identify true differences in brain volume across age groups. Longitudinal studies mitigate issues related to individual differences by allowing each participant to serve as their own control. There are fewer longitudinal studies on age-related brain atrophy, and they differ significantly in how participants are chosen and the techniques used (Raz et al., 2004; Fotenos et al., 2005; Jack et al., 2008). Longitudinal studies have documented annual declines in hippocampal volumes between 0.79% and 2.0% (Jack et al., 1998; Scahill et al., 2003; Raz et al., 2005; Du et al., 2006; Barnes et al., 2009; Ezekiel et al., 2004). Only few studies to date have incorporated both cross-sectional and longitudinal methodologies. The findings of these studies indicate that cross-sectional designs tend to underestimate the atrophy rates reported in longitudinal studies (See (Fraser et al., 2015) for a comprehensive review). However, longitudinal studies are also more resource-intensive, time-consuming, and susceptible to participant drop-out compared with cross-sectional designs. Given these complexities, it is essential to further investigate the relationship between cross-sectional and longitudinal measures of brain aging.

Moreover, neuroimaging research has demonstrated that hippocampal volumes are influenced not only by aging but also by various other conditions. Additional to age, factors such as female sex and APOE status are among the highest risk factors for sporadic AD and are therefore of high interest when studying hippocampal atrophy rates (Riedel et al., 2016). A study including over 36,653 healthy individuals found a significant interaction between age, sex, and APOE status affecting total hippocampal volume (Veldsman et al., 2021). Moreover, associations between Aβ accumulation and brain atrophy have been observed in individuals with MCI and AD (de Leon et al., 2006; Fjell et al., 2010b), as well as in cognitively normal older adults (Fjell et al., 2010a; Arenaza-Urquijo et al., 2013; Becker et al., 2011).

The goals of this longitudinal and prospective study of healthy elderly therefore were to (1) explore the relationship of a cross-sectional and longitudinal measurement of brain aging and (2) take into account potential influencing factors of hippocampal atrophy rates, such as APOE and amyloid status, education, as well as sex and (3) investigate the association with cognitive measures.

## 2 Materials and methods

### 2.1 Participants

This study utilized data from a large population-based longitudinal study on healthy aging (“Geneva Aging Study”). Study details have been described in prior publications (Montandon et al., 2020a; Xekardaki et al., 2015; Zanchi et al., 2017; van der Thiel et al., 2018). In short, healthy elderly participants with preserved cognition were recruited via advertisements in the local media of Geneva and Lausanne counties to ensure a community-based sample. Since a high level of French proficiency was necessary for the detailed neuropsychological evaluations, most participants (92%) were either Swiss or originated from French-speaking European countries. Exclusion criteria were as follows: present psychiatric or neurologic disorders, history of head injury, major medical disorders (neoplasm, stroke, or cardiac illness), alcohol or drug abuse, regular use of neuroleptics, antidepressants or psychostimulants, and contraindications to MR imaging. In total, 184 participants were enrolled in the study [110 females (59.8%) and 74 males (40.2%)]. All participants underwent structural brain Magnetic resonance imaging (MRI) at two different timepoints (mean period until follow-up: 4.8 ± 1.0 years). The mean age at baseline was 73.4 ± 3.5 years and the mean age at follow-up 78.1 ± 3.6 years. Education was assessed as an ordinal variable as a function of the formal years of training (< 9: obligatory, 9–12: high school, > 12: university).

The participants of the study underwent a comprehensive neuropsychological testing battery assessing different domains at baseline, 18-, and 54-months post-inclusion. The detailed list of the tests administered can be found in Table 1. Additionally, the Mini-Mental State Examination (MMSE; Folstein et al., 1975), the Hospital Anxiety and Depression Scale (HAD; Zigmond and Snaith, 1983), and the Lawton Instrumental Activities of Daily Living (IADL; Barberger-Gateau et al., 1992) were administered and all individuals were assessed using the Clinical Dementia Rating (CDR) scale (Hughes et al., 1982).

**TABLE 1:** Neuropsychological testing battery.

Domain	Test	Subdomain
Attention	Digit-symbol coding	
	Trail making test A	
Working memory	Digit span forward	verbal
	Visual memory span (corsi)	visuospatial
Episodic memory	Cued recall test	verbal
	Shapes test	visual
Executive function	Trail making test B	
	Wisconsin card sorting test and phonemic verbal fluency test	
Language	Boston naming test	
Visual gnosis	Ghent overlapping figures	
Praxis	Ideomotor skills	
	Reflexive skills	
	Constructional skills	

To capture both cognitive decline and improvement across various functions, a Continuous Cognitive Score (CCS) was established by converting all neuropsychological test results to z-scores. The number of tests showing an improvement of at least 0.5 standard deviation (SD) at follow-up (compared to baseline) was calculated (range: 0–14), as was the number of tests demonstrating a decline (range: 0–14). The final CCS was determined by subtracting the number of declined tests from the number of improved tests. Cognitive change between baseline and the final follow-up was defined as the sum of the CCS at 18 and 54 months (Montandon et al., 2020b).

### 2.2 MRI data acquisition

Imaging was performed at two timepoints at different scanners. At timepoint 1 (baseline), imaging was performed on a 3T MRI scanner (TRIO SIEMENS Medical Systems, Erlangen, Germany) and T1-weighted (T1w) data with the following parameters were acquired: 256 x 256 matrix, 176 slices, 1 mm isotropic, TR = 2.27 ms. At timepoint two, the follow-up, T1w data were acquired on a 3T MR750w scanner (GE Healthcare, Milwaukee, Wisconsin) with the following parameters: 254 x 254 matrix, 178 slices, 1 mm isotropic, TR = 7.24 ms. At both timepoints, additional sequences (T2-weighted imaging, susceptibility-weighted imaging, diffusion tensor imaging) were acquired to scan for incidental brain lesions. Only the high resolution T1w scans were used for this study.

### 2.3 MRI processing

All T1w images were visually inspected for quality. To extract brain regional volumes, the scans of each participant were processed by applying the longitudinal stream in FreeSurfer version 7.1.1 (Athinoula A. Martinos Center for Biomedical Imaging, Charlestown, MA, USA^1^) (Reuter et al., 2012). This longitudinal processing stream includes the creation of an unbiased within-subject template space and image (Reuter and Fischl, 2011) using robust, inverse consistent registration (Reuter et al., 2010). Next, skull stripping, Talairach transformation, atlas registration as well as spherical surface registration and parcellation are then initialized with common information from the within-subject template, which significantly increases reliability and statistical power (Reuter et al., 2012). Hippocampal volumes were extracted from subcortical segmentations for each subject. Estimated total intracranial volumes (ICV) were also calculated using the automated FreeSurfer preprocessing stream.

#### 2.3.1 Calculation of z-scores

All individual hippocampal volumes adjusted for age and ICV were transformed to z-scores by subtracting the mean of all hippocampal volumes of the study population and dividing by the SD. The residual method utilized in this study was described in detail in a prior publication (Opfer et al., 2022). The following equation was used to account for non-linear age effects on brain regional volumes (ROIV) according to Opfer et al. (2022):

ROIV=a×TIV+2b×age+2c×TIV×age+d×

T⁢I⁢V+e×a⁢g⁢e+f.

Subsequently, the residual of an individual ROIV estimate (resROIV) was calculated according to the following equation:

resROIV=ROIV-(a×TIV+2b×age+2c×TIV×

age+d×TIV+e×age+f)

In brief, in a first step, a linear regression was calculated with absolute hippocampal volumes as dependent and age at timepoint 1 as well as ICV as independent variables. The residuals of this regression were transformed into a z-score. Then, individuals with an age- and ICV-adjusted z-score of greater than +3 or less than −3 were considered as outliers and consequently removed (two individuals). Thus, the final sample consisted of 182 participants.

Next, the residual method was again applied to the absolute hippocampal volumes of the 182 individuals and age- as well as ICV-adjusted z-scores of the bilateral hippocampi were computed as described before to take into account that outliers might have influenced population mean and SD.

#### 2.3.2 Annualized percent change (APC)

To quantify the rate of hippocampal atrophy from baseline to follow-up, the annualized percent change (APC) was calculated for the absolute volumes of each of the bilateral hippocampi according to the following formula:

H⁢i⁢p⁢p⁢o⁢c⁢a⁢m⁢p⁢a⁢l⁢v⁢o⁢l⁢u⁢m⁢et⁢i⁢m⁢e⁢p⁢o⁢i⁢n⁢t⁢ 2-H⁢i⁢p⁢p⁢o⁢c⁢a⁢m⁢p⁢a⁢l⁢v⁢o⁢l⁢u⁢m⁢et⁢i⁢m⁢e⁢p⁢o⁢i⁢n⁢t⁢ 1H⁢i⁢p⁢p⁢o⁢c⁢a⁢m⁢p⁢a⁢l⁢v⁢o⁢l⁢u⁢m⁢et⁢i⁢m⁢e⁢p⁢o⁢i⁢n⁢t⁢ 1

$$*\frac{1}{t\,i\,m\,e\,i\,n\,t\,e\,r\,v\,a\,l}*100$$

### 2.4 Amyloid pet imaging

Among the sample a total of 103 participants also underwent amyloid PET imaging. Of these, 67 were obtained using 18F-Florbetapir (Amyvid) and 36 using 18F-Flutemetamol (Vizamyl). These scans were conducted on two different PET/CT systems, the Siemens Biograph mCT scanner and the GE Healthcare Discovery PET/CT 710 scanner, each with distinct resolution capabilities and acquisition protocols tailored to their respective platforms.

For the 18F-Florbetapir scans, imaging was performed 50–70 min post-injection, while the 18F-Flutemetamol scans were acquired 90–120 min post-injection. Image reconstruction followed the standardized ADNI protocol, designed to enhance consistency across multicenter acquisitions. Further details regarding the specific imaging procedures are available on the ADNI website^2^.

Amyloid status was visually assessed using standardized procedures endorsed by the European Medicines Agency (EMA). Additionally, all PET scans underwent intensity normalization using the thalamus-pons as the reference region, following the methodology described (Lilja et al., 2019). Subsequently, cortical standard uptake value ratios (SUVRs) were computed.

### 2.5 APOE-ε4 classification

APOE-ε4 status was determined following previously described methods (Zanchi et al., 2017). Participants were categorized based on their genotype, distinguishing between APOE-ε4 carriers and non-carriers (3/4, 3/3, and 2/3 genotypes).

### 2.6 Statistical analysis

Statistical analyses were performed using IBM SPSS Version 27 (IBM Corp., Armonk, NY, USA) as well as the STATA statistical software, Version 15.1 (StataCorp, College Station, Texas, 2017). One-tailed Pearson correlations were used to assess the association between z-scores of hippocampal volumes and rates of hippocampal atrophy (i.e., APC). To account for potential influencing factors, additional multiple regression analyses were computed including age, sex, education, and APOE as well as amyloid status as covariates. Since of the 182 participants only 103 underwent PET imaging, two different multiple regression models were computed: (1) including the information on amyloid status (*n* = 103) and (2) not including this information but controlling for all remaining variables (*n* = 182). This approach is appropriate as the amyloid data can be considered Missing Completely at Random (MCAR) based on the result of an MCAR test (*p* = 0.629). Consistent with the univariate analyses, one-tailed tests were performed to investigate the influence of baseline hippocampal volumes on hippocampal APC in the multiple regression, whereas for the remaining variables a two-tailed approach was used. Linear regression models with the CCSs as a dependent variable and z-transformed hippocampal volumes from baseline as independent variables were used to further explore the association with cognitive measures. Statistical significance was set as *p* < 0.05.

### 2.7 Ethics statement

The study was performed according to the declaration of Helsinki and approved by the local ethics committee. All subjects provided written informed consent prior to inclusion.

### 2.8 Data availability statement

The participant data used in this study are not publicly available but stored by the principal investigators of the Geneva Aging Study.

## 3 Results

**FIGURE 1:**
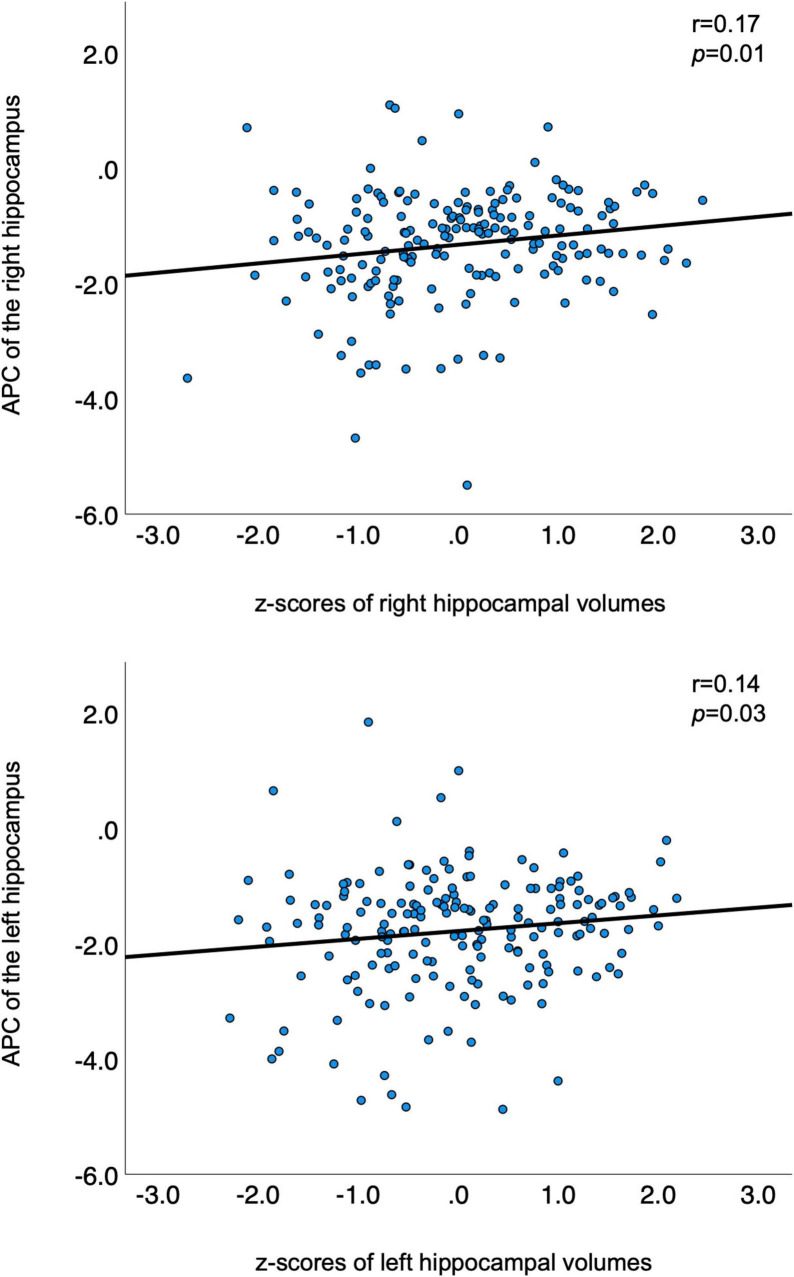
Correlations between APC and z-transformed volumes of bilateral hippocampi. APC is plotted on the *y*-axis; z-transformed volumes of the hippocampi on the *x*-axis. APC, annualized percent change.

Table 2 summarizes the demographic information of this cohort. The participants’ age at timepoint 1 ranged from 68 to 85 years and at timepoint 2 from 73 to 89 years. Left and right baseline hippocampal z-scores showed modest positive associations with APC of hippocampal volumes over time (see Figure 1, right hippocampus: *r* = 0.17, *p* = 0.01; left hippocampus: *r* = 0.14, *p* = 0.03). The lower the volume at baseline, the greater the greater the atrophy rate to follow-up. The mean volume of the right hippocampus at baseline was 3766.93 mm^3^ and at follow-up 3531.86 mm^3^ resulting in a mean APC of −1.34%. The mean volume of the left hippocampus at baseline was 3653.37 mm^3^ and at follow-up 3350.10 mm^3^ with a mean APC of −1.79%. Table 3 displays the mean values of bilateral hippocampal volumes, hippocampal z-scores at baseline, and APC of bilateral volumes of the hippocampus.

**TABLE 2:** Demographic characteristics.

Demographics	Healthy elderly participants (*n* = 182)
Mean age timepoint 1 (SD)	73.4 ± 3.5 years
Mean age timepoint 2 (SD)	78.1 ± 3.6 years
Sex	108 females (59.3%) and 74 males (40.7%)
Education	14.4% < 9 years (obligatory)
	45.6% 9–12 years (high school)
	40% more than 12 years (university)
APOE ε4 carriers	16.7%
Continuous cognitive score (SD)	−1.1 (4.1)

SD = standard deviation.

**TABLE 3:** Means of hippocampal volumes and APC.

Measure	Mean
Mean APC of the left hippocampus (SD)	−1.79 (1.00) %
Mean APC of the right hippocampus (SD)	−1.34 (0.94) %
Mean volume of the left hippocampus at TP 1 (SD)	3653.37 (378.72) mm^3^
Mean volume of the right hippocampus at TP 1 (SD)	3766.93 (398.73) mm^3^
Mean volume of the left hippocampus at TP 2 (SD)	3350.10 (389.78) mm^3^
Mean volume of the right hippocampus at TP 2 (SD)	3531.86 (420.14) mm^3^

SD = standard deviation, TP = time point.

In the regression model including information about amyloid status (*n* = 103) the effect of left z-transformed baseline hippocampal volumes on hippocampal APC was positive and statistically significant (left: β = 0.139, *p* = 0.047). Right hippocampal volume did not reach statistical significance within the subsample (β = 0.111, *p* = 0.12). None of other the added variables showed a statistically significant effect on the model (*p* > 0.05).

When excluding the information about amyloid status but including the remaining covariates and recalculating the model within the whole sample (*n* = 182) both left and right z-transformed baseline hippocampal volumes showed statistically significant effects on hippocampal APC (left: β = 0.131, *p* = 0.041; right: β = 0.162, *p* = 0.011). None of the other variables showed a statistically significant effect on the model (*p* > 0.05, please also see Table 4, 5).

**TABLE 4:** Regression model with right annual hippocampal atrophy (APC) as dependent variable.

Model (*n*)	Predictor	β (Coefficient))	SE	*t*	*p*	95% CI	R^2^	Adjusted R^2^	*F*	*p*
1 (182)	Baseline right HC volume	0.163	0.07	2.32	**0**.**011**	[0.024, 0.301]	0.04	0.004	1.13	0.345
	Sex	-0.044	0.15	-0.3	0.765*	[-0.335, 0.247]				
	Age	-0.021	0.02	-1.06	0.292*	[-0.061, 0.019]				
	9–12 y of education	0.036	0.21	0.17	0.862*	[-0.374, 0.446]				
	>12 y of education	0.066	0.21	0.31	0.759*	[-0.358, 0.489]				
	APOE4 status	-0.102	0.19	-0.53	0.595*	[-0.481, 0.276]				
	Intercept	0.22	1.5	0.15	0.883*	[-2.736, 3.176]				
2 (103)	Baseline right HC volume	0.111	0.09	1.18	0.12	[–0.076, 0.299]	0.03	–0.044	0.39	0.906
	Sex	-0.111	0.2	-0.57	0.573*	[–0.500, 0.278]				
	Age	-0.021	0.02	-0.83	0.407*	[-0.070, 0.029]				
	9–12 y of education	0.028	0.29	0.1	0.922*	[–0.542, 0.599]				
	− 12 y of education	0.104	0.3	0.34	0.732*	[–0.500, 0.709]				
	APOE4 status	0.003	0.26	0.01	0.990*	[–0.505, 0.511]				
	Amyloid status	0.067	0.22	0.3	0.764*	[–0.376, 0.510]				
	Intercept	0.249	1.85	0.13	0.893*	[–3.426, 3.924]				

CI, confidence interval, HC, hippocampus, SE, standard error, y, years, *, two-tailed *p*-value. Bold values indicate statistically significant results (*p* < 0.05).

**TABLE 5:** Regression model with left annual hippocampal atrophy (APC) as dependent variable.

Model (*n*)	Predictor	β (Coefficient)	SE	*t*	*p*	95% CI	R^2^	Adjusted R^2^	*F*	*p*
1 (182)	Baseline left HC volume	0.131	0.08	1.75	**0**.**041**	[-0.0169, 0.2781]	0.03	-0.003	0.92	0.484
	Sex	0.11	0.16	0.7	0.483*	[-0.1996, 0.4205]				
	Age	-0.007	0.02	-0.32	0.749*	[−0.0495, 0.0356]				
	9–12 y of education	-0.278	0.22	-1.25	0.211*	[-0.7146, 0.1592]				
	> 12 y of education	-0.151	0.23	-0.66	0.510*	[-0.6022, 0.3002]				
	APOE4 status	0.037	0.2	0.18	0.858*	[-0.3671, 0.4406]				
	Intercept	-1.157	1.6	-0.72	0.469*	[-4.3055, 1.9922]				
2 (103)	Baseline left HC volume	0.139	0.08	1.7	**0**.**047**	[-0.0236, 0.3008]	0.12	0.053	1.82	0.093
	Sex	0.316	0.19	1.69	0.094*	[-0.0553, 0.6866]				
	Age	-0.025	0.02	-1.06	0.290*	[−0.0721, 0.0218]				
	9–12 y of education	-0.359	0.27	-1.31	0.192*	[-0.9020, 0.1836]				
	> 12 y of education	-0.323	0.29	-1.11	0.270*	[-0.8989, 0.2539]				
	APOE4 status	0.242	0.24	1	0.321*	[-0.2395, 0.7229]				
	Amyloid status	-0.27	0.21	-1.30	0.198*	[-0.6839, 0.1439]				
	Intercept	0.247	1.76	0.14	0.889*	[-3.2491, 3.7433]				

CI, confidence interval, HC, hippocampus, SE, standard error, y, years, *, two-tailed *p*-value. Bold values indicate statistically significant results (*p* < 0.05).

Although all models account for only a modest proportion of variance (without amyloid status: left: R^2^ = 0.03, *p* = 0.48; right: R^2^ = 0.04, *p* = 0.34; including amyloid status: left: R^2^ = 0.118, *p* = 0.093; right: R^2^ = 0.028, *p* = 0.91), R^2^ of the left hippocampus increases when including amyloid status. Therefore, the inclusion of amyloid status appears to attenuate the association between hippocampal volume and APC.

There was no statistically significant relationship between z-transformed hippocampal volumes from baseline and the CCSs (right hippocampus: *p* = 0.11, left hippocampus: *p* = 0.12; Supplementary Figure 1).

## 4 Discussion

The goals of this longitudinal study of healthy elderly were to (1) explore the relationship of a cross-sectional and longitudinal measurement of brain aging, namely the association between baseline hippocampal volumes and APC in hippocampal volumes over time, and (2) take into account potential influencing factors of hippocampal atrophy rates, such as APOE and amyloid status, education, as well as sex and (3) investigate the association with cognitive measures. We found modest, but statistically significant correlations between baseline hippocampal z-scores and APC of hippocampal volumes for both hemispheres, indicating that the lower the volume at baseline, the greater the atrophy rate to follow-up. APOE and amyloid status, education, and sex did not show a statistically significant influence on this relationship in this sample.

### 4.1 Normal aging and atrophy rates

Medial temporal lobe structures, such as the hippocampus, are of specific interest when studying aging since this is the first region of the brain affected by the neurofibrillary pathology of AD [for review see (Pini et al., 2016)]. Recent studies on age-related atrophy showed that atrophy rates vary across the cognitive continuum (Barnes et al., 2009; Jack et al., 1998). Previous studies reported differences in hippocampal APC in healthy controls, patients with MCI, as well as AD with most pronounced values in AD (Jack et al., 2000). In this study of healthy elderly participants, we found a decrease in hippocampal volumes from baseline to follow-up. Numerous studies have investigated typical brain aging and reported different annual atrophy rates (Fraser et al., 2015). The APC in this sample was 1.79% for the left and 1.34% for the right hippocampus, with an average of 1.57% of both sides. A meta-analysis by Barnes et al. (2009) reported an average APC of 1.41%, while another more recent meta-analysis by Fraser et al. (2015) found an average of 1.12% for those aged 70 and above. The APC in this study is similar to Barnes et al.’s (2009) and slightly higher than the average reported by Fraser et al.’s (2015). However, the APCs reported in the appropriate age subgroup of age 70 and older ranged from 0.32 (Driscoll et al., 2009) to 2.34% (Wang et al., 2003), which concurs with the 1.57% APC found in this sample. Interestingly, the left hippocampus showed an increased APC compared with the right hippocampus in this sample. There is evidence that the left hippocampus undergoes more rapid volumetric changes compared with the right hippocampus in the literature. For example, Thompson et al. (2003) found that the local left hemispheric gray matter shows a faster rate of atrophy compared with the right side in both patients with AD and healthy controls. However, a meta-analysis by Minkova et al. (2017) did not find laterality effects in healthy aging.

### 4.2 Fit of atrophy rate

Substantial evidence indicates that hippocampal volumes do not decline linearly but rather show a non-linear decrease throughout adulthood (Vinke et al., 2018; Nobis et al., 2019; Bethlehem et al., 2022; Bakhtiari et al., 2023; Fjell et al., 2013). Previous research showed that hippocampal volumes remain relatively stable in young adulthood. However, after a certain age point, there appears to be a critical inflection point where the rate of atrophy accelerates significantly, reaching an annual decline of about 0.8%–0.9% (Nobis et al., 2019; Fjell et al., 2009). Beyond this threshold, hippocampal volumes continue to decrease progressively with advancing age. To acknowledge these non-linear age-related effects, a quadratic age term was used to adjust baseline hippocampal volumes in this study (Opfer et al., 2022). The hippocampal atrophy rates in our study were calculated by a linear fit applied to two time points, primarily for reasons of practicality and simplicity. While this approach is straightforward, it does not fully capture the complexity of hippocampal volume changes over the lifespan. While our use of a linear model to calculate atrophy rates is methodologically justified for this study’s purposes, future work could benefit from incorporating more sophisticated modeling approaches to better capture the dynamics of hippocampal atrophy across the adult lifespan.

### 4.3 Longitudinal and cross-sectional measurements

Serial MRI studies permit calculation of atrophy rates over time. While our study is a longitudinal study, it focused on an association between a cross-sectional and a longitudinal measure using hippocampal volume measurements. Both, cross-sectional and longitudinal imaging studies have found a correlation between increasing age and decreasing hippocampal volumes (Mueller et al., 1998; Resnick et al., 2000; Coffey et al., 1992; Mu et al., 1999). However, evidence exists that the reported rates of atrophy tend to differ depending on the study design (Fraser et al., 2015). Only a few studies to date have directly compared cross-sectional and longitudinal measures of atrophy rates (Du et al., 2006; Ridha et al., 2006; Scahill et al., 2003). Current findings remain mixed, with cross-sectional studies tending to underestimate age-related effects. For example, Du et al. (2006) investigated 42 cognitively healthy elderly participants aged 58 to 87 years and identified a significant aging effect on hippocampal atrophy longitudinally, but not cross-sectionally. Another study by Ridha et al. (2006) included serial MRI among 9 patients with an autosomal dominant mutation for AD (3–8 scans per patient) and 25 cognitively healthy controls (2–4 scans per control). In their study, longitudinal measurement of hippocampal and whole brain atrophy rates detected differences between mutation carriers and controls 2–3 years before cross-sectional assessments. Raz et al. (2005) investigated 140 cognitively healthy participants of which 127 completed a longitudinal follow-up. Hippocampal atrophy rates measured cross-sectionally were considerably lower compared with longitudinal measurements. However, a more recent study by Fjell et al. (2013) examined age-trajectories of several subcortical volumes including the hippocampus of 1100 individuals cross-sectionally and 142 longitudinally. They reported a fairly consistent relationship between the atrophy patterns observed in the cross-sectional and longitudinal results (Spearman’s ρ = 0.91) (Fjell et al., 2013).

In this study, we found a modest relationship between the cross-sectional and longitudinal hippocampal atrophy pattern: the smaller the hippocampal volume at baseline, the higher the annual atrophy rate. Thus, combining both study designs could provide a more comprehensive understanding of hippocampal atrophy and its relationship to aging. This could also be an important finding for the interpretation of hippocampal atrophy in clinical practice: As the term “atrophy” means a loss of previously present tissue, our study suggests that baseline hippocampal volumes also need to be considered when determining whether hippocampal volume loss is increased or not. Also, as lower hippocampal volumes are associated with increased rates of hippocampal volume loss, maybe the threshold to identify decreased hippocampal volume in cross-sectional studies needs to be adapted. This should be tested in larger population studies. The modest magnitudes of the correlations, however, also suggest that, while baseline hippocampal volume may serve as one predictor of future atrophy, other factors, such as genetic, lifestyle, or additional biomarkers, may likely contribute substantially to individual variability in degeneration rates.

### 4.4 Associations with potential influencing factors and cognition

We did not find a statistically significant relationship between hippocampal volumes from baseline and the CCS. This may reflect the fact that the sample consists of healthy elderly individuals who do not experience cognitive complaints.

Further, in this sample none of the investigated covariates, APOE and amyloid status, education, nor sex showed a statistically significant influence on the association between hippocampal APC and baseline hippocampal volumes. In a previous study of a subgroup of this sample (*n* = 81) APOE genotype showed a statistically significant effect on hippocampal volume loss (Haller et al., 2019). However, in this larger sample, this relation was not statistically significant. The studies investigating the effect of the APOE-ε4 allele on brain structure in healthy elderly individuals remain mixed. While some studies have reported a link between the APOE-ε4 allele and reduced hippocampal volumes (Haller et al., 2019; Veldsman et al., 2021), others have not (Gonneaud et al., 2016; Squarzoni et al., 2018). Future studies are needed to investigate this association further.

Interestingly, the inclusion of amyloid status appeared to attenuate the association between hippocampal volume and APC. This was most notable for the left hippocampus, where R^2^ increased from 0.03 to 0.12. Correlations between hippocampal volumes and Aβ accumulation have been reported previously (Mormino et al., 2009; Arenaza-Urquijo et al., 2013; Fjell et al., 2014), however, when compared to associations with other subcortical structures, the relationship between Aβ and hippocampal atrophy did not appear to be particularly pronounced (Fjell et al., 2014; Fjell et al., 2010a). In a previous investigation of this sample, the effect of amyloid status on hippocampal atrophy was at the level of a (statistically non-significant) trend (Haller et al., 2019). The results found in this study are similar. The findings suggest that amyloid accumulation may impair hippocampal integrity only in a subset of particularly vulnerable individuals (Nosheny et al., 2015; Duarte-Abritta et al., 2018; Khan et al., 2017; Haller et al., 2019). It is worth noting, however, that only a small proportion of the sample was amyloid positive (24.3% of the 103 participants who underwent PET scanning). Therefore, this association should be investigated further in larger samples.

It is known that female sex is one of the strongest risk factors of AD. Previous research has shown, that female APOE-ε4 carriers with MCI experience more pronounced hippocampal volume loss and memory decline compared to male APOE-ε4 carriers (Veldsman et al., 2021). In this sample of healthy elderly female sex, however, did not show a statistically significant influence. Among the reasons may be that only cognitively normal individuals were included in this investigation and even subjective cognitive complaints were a strict exclusion criterion. Moreover, volumes were controlled for ICV as a partial correction for sex which may have precluded statistically significant findings (Scahill et al., 2003; Dekaban, 1978). Similar reasons may explain that education showed no statistically significant effect, while it is known that greater cognitive reserve, reflected in factors such as education level and socio-economic status, has been shown to reduce the risk of AD (Stern, 2012).

### 4.5 Limitations

A key limitation of the current study is that the imaging data were acquired on two different scanners at timepoint 1 and timepoint 2. Specifically, a 3T SIEMENS scanner was used at timepoint 1, while a 3T GE Healthcare scanner was used at timepoint 2, which could potentially introduce systematic errors. Of note, a study by Brown et al. (2020) evaluated the test-retest reliability of the FreeSurfer automated hippocampal subfield segmentation procedure using two Siemens model scanners and reported high reliability both within and between scanners. Another study by Quattrini et al. (2020) investigated the test-retest reliability of hippocampal subfields in a sample of healthy elderly subjects scanned at 13 different clinical sites. They found that larger volumes showed the most reliable test-retest results, with hippocampal subfields over 300 mmł achieving ε < 5% and DICE > 0.80. While we used a recent FreeSurfer longitudinal pipeline (v7.1.1), we acknowledge that alternative segmentation methods such as MUSE (Srinivasan et al., 2020) or LASHiS (Shaw et al., 2020) may offer similar or improved accuracy or consistency, particularly across sites or in subfield analyses, and should be explored in future studies.

Also, individuals who participate in research studies (such as the Geneva Aging study) may not fully represent the general clinical population. Future studies could expand the dataset by including brain scans collected from regular clinical visits.

## 5 Conclusion

The findings of this study suggest that hippocampal z-scores obtained cross-sectionally may predict future hippocampal volumetric changes.

## Data Availability

The datasets presented in this article are not readily available because they are stored by the principal investigators of the Geneva Aging Study. Requests to access the datasets should be directed to VS, vivian.schultz@tum.

## Footnotes

1
http://surfer.nmr.mgh.harvard.edu

2
http://adni.loni.usc.edu/methods/

## Supplementary material

Supplementary Figure 1 Association of baseline hippocampal z-scores and cognitive measures.
