# Supplementary figures and images for: Lower hippocampal volumes at baseline are associated with higher volume loss in healthy elderly
Source: Front Aging Neurosci. 2025 Jul 16;17:1542857. doi: 10.3389/fnagi.2025.1542857 (PMC12307302; doi:10.3389/fnagi.2025.1542857)

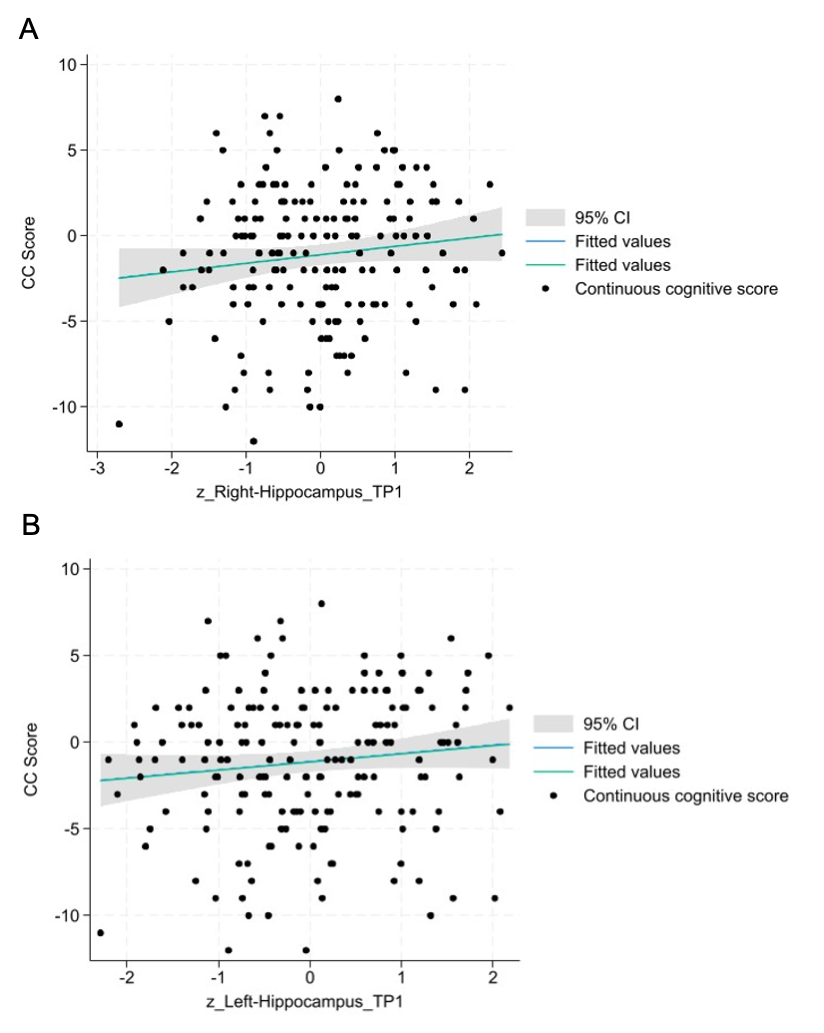

Supplement: Supplementary Figure 1 — Association of baseline hippocampal z-scores and cognitive measures. [file Image_1.png]
